# Two rare mutations in homozygosity synergize to silence TREX1 in Aicardi-Goutières syndrome

**DOI:** 10.3389/fimmu.2025.1557632

**Published:** 2025-02-21

**Authors:** Tamar Rubin, Stéphane Bernier, Lily Siok Hoon Lim, Michael S. Salman, Edward Leung, Aziz Mhanni, Sandra Marles, Cheryl Greenberg, Anna Perez, Yichun Sun, Isabelle Angers, Donald C. Vinh, Lucie Roussel

**Affiliations:** ^1^ Section of Pediatric Clinical Immunology and Allergy, Department of Pediatrics and Child Health, University of Manitoba, Winnipeg, MB, Canada; ^2^ IMMUNO-GRAM (Infection and IMMunity Genetic Research to Advance Molecular Medicine) Center of Reference, Research Institute - McGill University Health Centre, Montreal, QC, Canada; ^3^ Section of Pediatric Rheumatology, Department of Pediatrics and Child Health, Children's Hospital Research Institute of Manitoba, University of Manitoba, Winnipeg, MB, Canada; ^4^ Section of Pediatric Neurology, Department of Pediatrics and Child Health, University of Manitoba, Winnipeg, MB, Canada; ^5^ Section of Genetics and Metabolism, Department of Pediatrics and Child Health, University of Manitoba, Winnipeg, MB, Canada; ^6^ Department of Medicine (Division of Infectious Diseases), McGill University Health Centre, Montreal, QC, Canada; ^7^ Department of OptiLab (Division of Medical Microbiology, Division of Molecular Genetics-Immunology), McGill University Health Centre, Montreal, QC, Canada; ^8^ Department of Human Genetics, McGill University, Montreal, QC, Canada

**Keywords:** Aicardi-Goutières syndrome (AGS), type I interferon, TREX1 gene, autoinflammatory disorders, cGAS-STING pathway, Inuit population

## Abstract

**Background:**

Aicardi-Goutières syndrome (AGS) is a rare monogenic type I interferonopathy characterized by dysregulated inflammation and tissue damage that primarily affects the central nervous system. AGS is genetically diverse, with pathogenic variants across multiple genes, including TREX1, which drives excessive type I interferon (IFN) production.

**Objective:**

This study investigated the genetic and molecular mechanisms underlying AGS in a family of two affected children, focusing on the role of *TREX1* variants in protein expression and dysregulation of the interferon pathway.

**Methods:**

Genomic sequencing data were used to identify *TREX1* variants in the affected children. Functional assays in patient-derived lymphoblastoid cells (LCLs) and cell line models were used to evaluate TREX1 expression and activation of the cGAS-STING pathway.

**Results:**

Two homozygous *TREX1* variants were identified in two affected children. Functional analyses showed that both variants are required to mirror the near-absent protein levels observed in LCL and to cause excessive activation of IRF3 in cGAS-STING pathway in response to cytosolic DNA stimulation.

**Conclusion:**

To our knowledge, our findings demonstrate, for the first time, the compound effect of two rare homozygous variants account for AGS. This also reiterates the importance of molecular and functional assessments of genomic variants identified by sequencing.

## 1 Introduction

Aicardi-Goutieres syndrome (AGS) is a rare genetic disorder typically presenting within the first year of life, characterized by chronic, severe neurological impairment, intracranial calcifications, and immunological dysfunction, as well as variable other manifestations (e.g. chilblain skin lesions) (1). Common biomarkers include elevated IFN-α levels in cerebrospinal fluid and blood, along with increased type I IFN-stimulated gene (ISG) expression in whole blood (2–4). The syndrome, “Cree encephalitis”, first described in 1984, is allelic to AGS (5). To date, variants in nine genes underlie AGS (REX1, RNASEH2A, RNASEH2B, RNASEH2C, SAMHD1, ADAR1, IFIH1, LSM11 et RNU7-1) (6, 7). The identification of these variants, as well as their translation into pathogenic type I interferon signaling activity stand as landmark evidence defining AGS as a type I interferonopathy. To date, variants in seven genes underlie AGS. The identification of these variants, as well as their translation into pathogenic type I interferon signaling activity stand as landmark evidence defining AGS as a type I interferonopathy (8).

Among the genes causing AGS is Three Prime Repair Exonuclease 1 (*TREX1*). Variants in *TREX1* cause various syndromes including those associated with excessive type I interferon signaling include autosomal-recessive (AR) Aicardi-Goutières syndrome; autosomal-dominant (AD) AGS; AD familial chilblain lupus (FCL); and AD systemic lupus erythematosus (SLE) (9). Genetic lesions of TREX1 also underlie AD Retinal vasculopathy with cerebral leukodystrophy (RVCL) (10), which is apparently not associated with the prototypical type I interferonopathy signature (11).

TREX1 encodes a highly-abundant, mammalian 3’-to-5’- DNA exonuclease involved in genomic DNA degradation, repair, and proofreading (12–14). TREX1 is also involved in immune regulation, notably of the cyclic GMP-AMP synthase (cGAS), a stimulator of interferon genes (STING) pathway (15, 16). Double-stranded DNA binds to cGAS, resulting in synthesis of cGAMP, which then binds and activates STING (15, 17, 18). Activation of STING leads to the recruitment and activation of Tank Binding Kinase 1 (TBK1) and interferon regulatory factor 3 (IRF3), with the latter dimerizing, translocating to the nucleus, and stimulating the transcription of type I interferons (e.g. interferon-alpha, interferon-beta) and other interferon-stimulated genes (ISG) (19–21). Cytoplasmic TREX1 normally degrades DNA substrates that serve as agonists of the cGAS-STING pathway, mitigating the interferon response (22, 23).

*TREX1* is a single-exon gene that encodes for three transcripts of variable length at the N-terminus (14). Most damaging variants of *TREX1* underlying AR AGS are either missense or frameshift, causing decreased exonuclease activity (Loss-of-function, LOF) (10, 14, 24, 25). Here, we report two pediatric siblings, each carrying two homozygous variants in the 5’ region of *TREX1*, and demonstrate that the concomitant presence of both variants leads to near-complete absence of expression, with LOF, expanding the genomic spectrum by which AR AGS may develop.

## 2 Materials and methods

### 2.1 Human subjects

Written informed consent was obtained by all patients or their legally authorized representative. The study was approved by the McGill University Health Centre Research Ethics Board protocol 10-256.

### 2.2 Sequencing and bioinformatics analysis

For Sanger sequencing, the *TREX1* gene was PCR amplified from genomic DNA using primers designed to flank the respective regions (primers and sequencing conditions available on request). Sequencing was performed at the Génome Québec Innovation Centre (Montreal, Canada). Sequencing analyses were performed on Sequencher^®^ sequence analysis software (Gene Codes Corporation, Ann Arbor, MI).

### 2.3 Cell culture

Epstein-Barr virus (EBV)-induced lymphoblastoid cell lines (LCL) derived from peripheral blood mononuclear cells (PBMC) were cultured in RPMI supplemented with 10% fetal bovine serum (FBS), 20mM HEPES and penicillin/streptomycin (100U/ml and 100ug/ml respectively (Wisent). Fibroblasts (IMR-90) were purchased from ATCC and were cultured in EMEM supplemented with 10% fetal bovine serum (FBS) and penicillin/streptomycin (100U/ml and 100ug/ml respectively; Wisent).

### 2.4 Antibodies and stimulation

TREX1 polyclonal antibody was from Proteintech. Anti-GAPDH was from Millipore. Secondary antibodies for immunoblotting: DyLight800-anti-rabbit-IgG and DyLight680-anti-mouse-IgG were from ThermoScientific. Alexa Fluor 647 anti-IRF3 and PE phospho-IRF3 (Ser396) were from Cell Signaling Technology. Poly(dA:dT) and Poly(dG:dC) were from Invivogen. IFN-alpha was from PBL Assay Science.

### 2.5 DNA constructs and transfection

The myc-flag tagged wild-type (WT) *TREX1* plasmid was purchased from OriGene (RC214869) and confirmed WT by Sanger sequencing. The WT TREX1 plasmid served to create both variants using site-directed mutagenesis kit (New England Biolabs). Liposomal transfection in fibroblast cells was performed with the Lipofectamine 3000 Transfection Reagent (Invitrogen) per the manufacturer’s instructions. Cells were harvested 48 hours after transfection.

### 2.6 Immunoblotting

LCL were resuspended in RIPA lysis buffer (Sigma) supplemented with complete Mini Protease Inhibitor Cocktail and PhosSTOP (both from Roche), then boiled in LDS Sample buffer and Blot Sample Reducing Agent (Thermo Fisher Scientific). Protein was separated by SDS-PAGE on premade gels (Novex) before being transferred to a membrane via iBlot Gel Transfer Device (Invitrogen). Membrane was blocked in 5% skim milk TBST to block nonspecific protein binding. The membrane was blotted using primary antibodies overnight at 4°C. The membrane was washed 4 times with 0.1% Tween 20 in TBS and then incubated with a 1:15,000 dilution of secondary antibodies in blocking buffer. Membranes were scanned and analyzed with an Odyssey IR scanner using Odyssey imaging software 3.0 (LI-COR Biosciences, Inc).

### 2.7 Quantitative real-time PCR and interferon-stimulated gene score calculation

RNA from PBMC was extracted using Trizol Reagent (Invitrogen) per manufacturer’s instructions and quantified by NanoDrop-100 spectrophotometer. 500ng of RNA was reverse transcribed using the Maxima H Minus First Strand cDNA Synthesis Kit (Thermo Fisher Scientific). Real-time quantitative PCR was performed with CFX Opus 96 Real-Time PCR (Bio-Rad Laboratories). TaqMan quantitative PCR assays were run using primers/probes: *TREX1* (Hs03989617), *IFI27* (Hs01086370), *SIGLEC1* (Hs00988063), *IFL44L* (Hs00199115), *MX1* (Hs00895608), *RSAD2* (Hs01057264), *ISG15* (Hs01921425) and *IFIT1* (Hs00356631). The mRNA input was normalized to the expression of the housekeeping genes, *GAPDH* (Hs02786624) or *HPRT1*(Hs03929096).

ISG scores were calculated for healthy control (HC) and AGS patients (P1 and P2) at multiple time points following IFNα stimulation (2h, 4h and 24h). Quantitative PCR was conducted for a panel of seven ISGs: [*IFI27, IFI44L, IFIT1, ISG15, MX1, RSAD2 OAS1, SIGLEC1*]. ISG scores were then calculated as the median of the normalized expression values for the seven ISGs for each sample and time point (26).

### 2.8 Flow cytometry

Cells were blocked in Fc Receptor Binding Inhibitor Monoclonal Antibody (Invitrogen) and incubated with LIVE/DEAD Fixable Dead Cell Stain (Thermo Fisher Scientific). For intracellular staining, cells were stained using the FIX & PERM Cell Fixation and Cell Permeabilization Kit (Thermo Fisher Scientific). Samples were analyzed using 4-laser Cytek Aurora flow cytometer (Cytek Biosciences). Data analyses were performed using FlowJo (Ashland, OR). For statistical analysis, repeated-measures one-way ANOVA, followed by Tukey’s multiple comparison post-test were used. The level of significance was set at *p<0.1234, **p<0.0332, ***p<0.0021, ****p<0.0001.

## 3 Results

### 3.1 Case description

**Figure 1:**
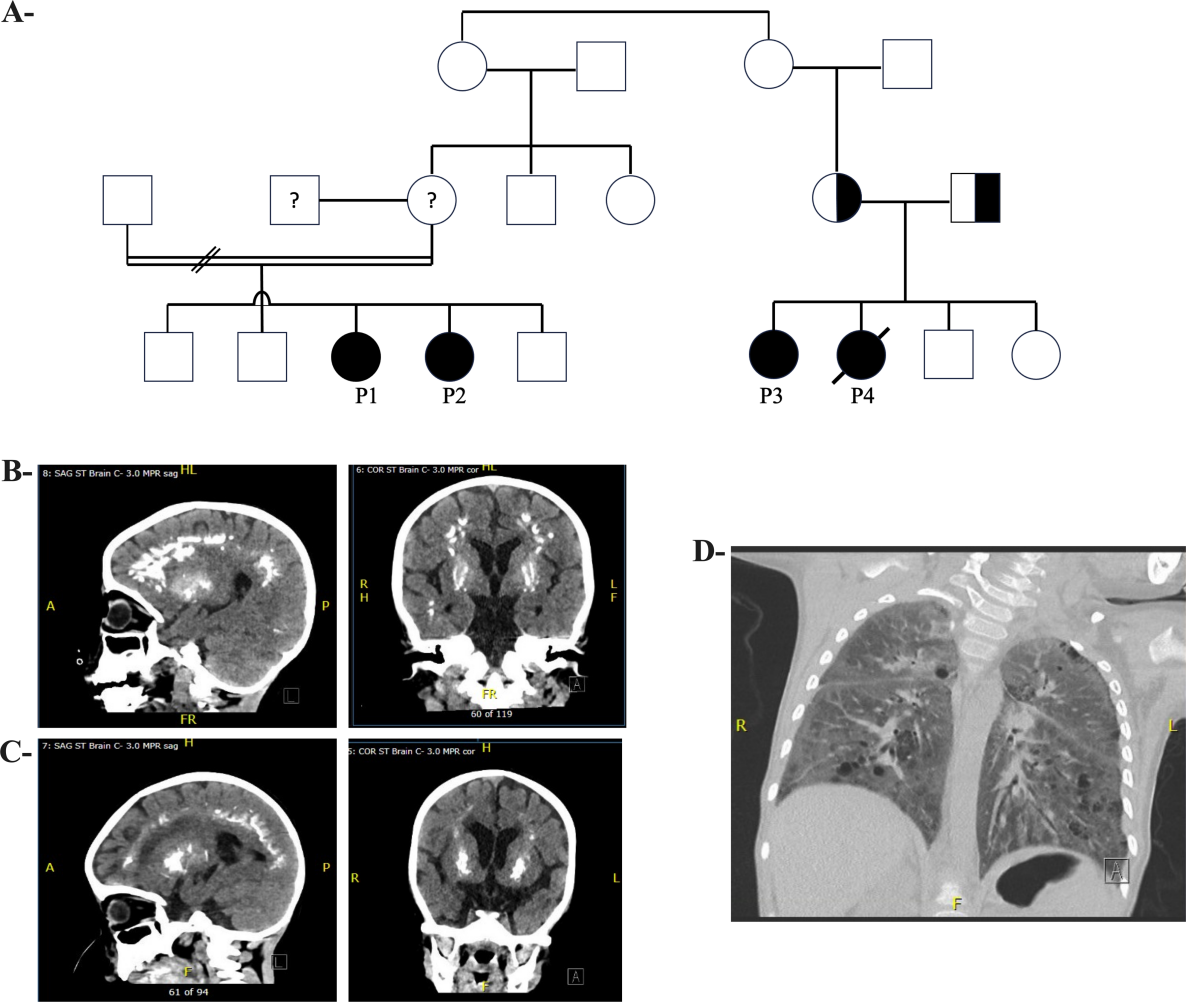
Pedigree and Radiological Features of Patients with TREX1 Variants **(A)** Pedigree showing family of Patients 1 and 2 (I.) and Patients 3 and 4 (II.). **(B)** (Left) Sagittal brain CT and (Right) Coronal brain CT of Patient 1, at 3.5 years old, with calcification involving subcortical white matter of bilateral cerebral hemispheres, as well as centered around bilateral putamen. **(C)** (Left) Sagittal brain CT and (Right) Coronal brain CT of Patient 2, at 26 months old, with symmetric, linear, arch and flame-shaped calcifications near gray-white junction/subcortical white matter throughout bilateral frontal parietal and occipital lobes, and rounded opacifications within basal nuclei. **(D)** CT chest of Patient 1 with diffuse bilateral ground glass opacification and interlobular septal thickening.

Patients 1 (P1) and 2 (P2) are siblings, born to endogamous Inuit parents (**Figure 1A**; see **Supplementary Information** for full clinical details). P1 had gross motor developmental delay, hypotonia (e.g., no head control at 5 months old, inability to roll over at 10 months of age), and chronic constipation. At 21-months old, neurologic and developmental assessments revealed marked global developmental delay, with developmental skills at the 10-month level and gross motor skills at the 4-to-5-month level. MRI of the brain at 21 months revealed symmetric curvilinear and amorphous areas of increased susceptibility-weighted imaging (SWI) signal intensity involving the cortex and subcortical white matter in the frontal, parietal, and temporal lobes, suggesting hemosiderin or calcification. Additionally, subtle, symmetric hyperintensities were observed within the deep grey structures on SWI. CT imaging showed bilateral symmetrical calcification in the subcortical white matter and branch-like calcifications within the globus pallidus bilaterally (**Figure 1B**). Chromosomal microarray demonstrated multiple regions of homozygosity, attributed to consanguinity, but was otherwise unremarkable. Metabolic testing and fragile X screening were negative. P1 developed recurrent pneumonitides starting at age 1, attributed to aspiration and viral respiratory infections. At age 3, she was frequently admitted to the Pediatric Intensive Care Unit (PICU) for pneumonia with respiratory failure, despite efforts to prevent oropharyngeal aspiration with exclusive nasogastric tube feedings. Mild hepatosplenomegaly was noted. Immunologic workup for these recurrent pneumonias revealed mild chronic anemia, hypergammaglobulinemia, and a mildly increased proportion of alpha/beta-TCR double negative T cells, in the context of mild lymphopenia. Despite being fully immunized (vaccines administered up to age 2, including pneumococcal polysaccharide vaccine), titers were undetectable for varicella, measles, and rubella; diphtheria titers were 0.05 IU/mL (indeterminate immunity); and tetanus titers were 0.11 IU/mL (suggesting short-term immunity). Pneumococcal titers were low. However, 4 weeks after boosters with MMRV, pneumococcal conjugate, and DTaP-IPV-Hib vaccines, P1 showed a robust immune response. P1 also had low-level Epstein-Barr virus (EBV) viremia (3.61x 10^3 copies/mL). Repeat CT imaging of her brain showed progressive calcification of the subcortical white matter and cerebral volume loss (**Figure 1B**). Based on genetic and molecular investigations, it was suspected that her repeated admissions with pneumonic consolidations and respiratory failure may be due to alveolar hemorrhage or dysregulated inflammation, rather than infection. She was started on pulse corticosteroids, with improvement. The family declined treatment with baricitinib (a JAK1/2 inhibitor).

P2 was noted to have delayed milestones from 3 months of age. By 7 months, the patient was hospitalized for a pneumonic process, and brain CT and MRI showed bilateral calcifications in the lenticulostriate regions (**Figure 1C**). A TORCH screen was positive for HSV-1 IgM and IgG. P2’s course was marked by recurrent hospitalizations for respiratory failure suspected to be aspiration pneumonia (**Figure 1D**), developmental delay, hypotonia with hypertonic extremities, progressive loss of milestones, and epilepsy. At 2 years old, an MRI showed new calcifications, delayed myelination, demyelination, and atrophy of the frontal and temporal lobes. P2 was genetically assessed at 7 months old, including biochemical metabolic tests and chromosomal microarray, but no diagnosis was established. A whole exome sequencing (WES) was also performed, but was non-diagnostic. Immunologic investigations revealed that P2 did not generate protective responses to a single dose of MMRV vaccine and had low titers for other vaccines after initial immunization (**Table 1**). The family was offered investigational baricitinib treatment, which they declined.

**Table 1:** Vaccine titers and response 4 weeks after booster MMRV, DTaP-IPV and 23-valent pneumococcal polysaccharide immunizations for Patients 1, 2, 3.

Vaccine Titers	Patient 1	Patient 2	Patient 3
Measles IgG (4 weeks post-booster)	**Not detected** (detected)	**Not detected**	Detected
Mumps IgG (4 weeks post-booster)	Detected (detected)	**Not detected**	**Not detected**
Rubella IgG (4 weeks post-booster)	**Not detected** (detected)	**Not detected**	**Not detected**
Varicella IgG (4 weeks post-booster)	**Not detected** (detected)	**Not detected**	Not done
Tetanus toxin IgG (4 weeks post-booster)	**0.111 IU/mL** (>5 IU/mL)	**0.111 IU/mL**	**0.127 IU/mL**
Diphtheria toxin IgG (4 weeks post-booster)	**0.050 IU/mL** (>2 IU/mL)	Not done	1.476 IU/mL
Streptococcus pneumoniae capsular polysaccharide IgG (4 weeks post-booster)	**10.273 mg/L** (262.462 mg/L)	40.677 mg/L	29.888 mg/L
Streptococcus pneumoniae capsular polysaccharide IgG2 (4 weeks post-booster)	**1.782 mg/L** (22.349 mg/L)	10.181 mg/L	10.634 mg/L
Serotype-specific pneumococcal response	Protective response to 18/22 serotypes (and 6/10 serotypes exclusively in Pneumovax)	Not done	Not done

Bold values are outside the normal range.

Family history revealed P1/P2’s paternal uncle and maternal second cousin also had a child with global developmental delay and intracranial calcifications (P3), as well as a deceased child at age 1. This prompted genetic testing in P1 and re-evaluation of P2’s WES data.

### 3.2 Genetic evaluation

**Figure 2:**
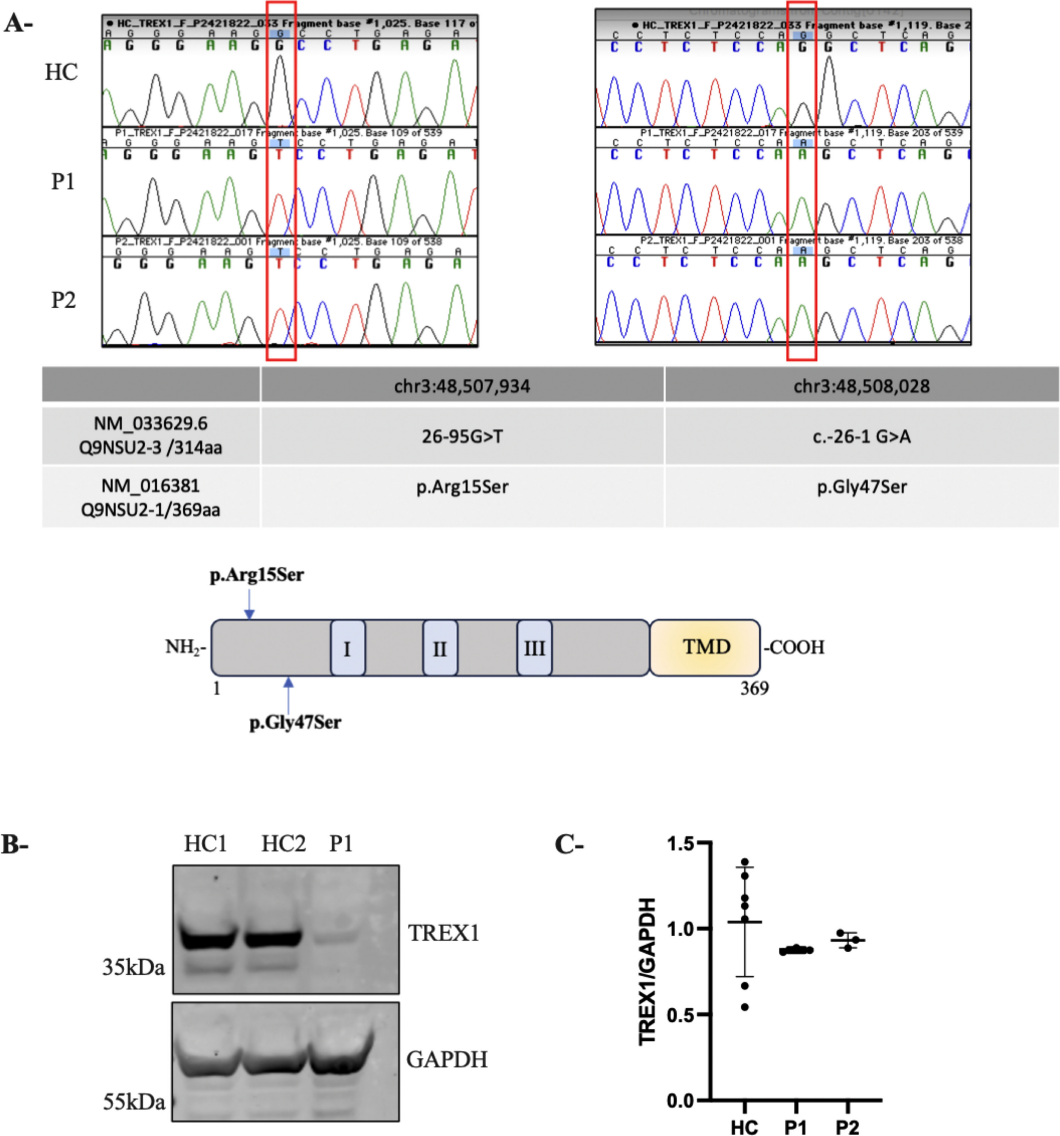
Novel homozygous *TREX1* variants leading to TREX1 deficiency. **(A)** Chromatogram of Sanger sequencing from a healthy control (HC) and 2 patients AGS (P1 and P2) showing the corresponding mutations in *TREX1.* The table represents the two isoforms of TREX1 relevant to this study. A schematic TREX1 protein. TREX1 has three exonuclease domains (I, II, III) and a putative transmembrane domain (TMD). **(B)** Immunoblotting showing TREX1 expression in whole-cell lysates from subject-derived LCL (2 healthy control (HC1, HC2) and AGS patient (P1); GAPDH served as a loading control. **(C)** Expression of *TREX1* as determined by qRT-PCR in PBMC from patients. The values represent the mean +/- SEM fold change in expression relative to housekeeping gene (*GAPDH*). Data are representative of three independent experiments.

Initial genetic analysis of P1 revealed a homozygous G>A variant at chr3:48,508,028 in the *TREX1* gene, designated as c.-26-1G>A, based on the reference isoform annotated in the UCSC genome browser (GRCh37/hg19) and NCBI (NM_033629.6), or as p.G47S (NM_016381) (**Figure 2A**). This variant has been previously documented in the gnomAD database (minor allele frequency, MAF: 20/281,302; 7.11×10^-5) though it has not been observed in the homozygous state. The G>A substitution affects a conserved nucleotide in a canonical splice acceptor site and is predicted to disrupt mRNA splicing and consequently alter the gene product. This variant was similarly identified in P2. Genotyping was not possible for their parents and extended family members. P1 and P2 were also found to carry a homozygous chr3:48,507,934 G>T variant upstream of the initially identified one, designated as c.-26-95G>T (NM_033629.6) or as p.R15S (NM_016381) (**Figure 2A**). The variant R15S is reported in gnomAD with a MAF of 112/278476 (4.02e-4), including two homozygotes, and is reported as ‘likely benign’. Given the AGS-like phenotype of the two children, further characterization of the variants was pursued.

### 3.3 Functional validation

To determine the impact of the two variants on TREX1 expression, we performed immunoblot on lysates from LCLs derived from P1 and healthy controls (HC). Due to limited availability of patient material, we were unable to generate an LCL for P2. Normalization of protein levels to GAPDH expression revealed a near-complete absence of TREX1 protein in P1, confirmed using both monoclonal (data not shown) and polyclonal antibodies (**Figure 2B**). In parallel, RT-PCR analysis on RNA extracted from PBMCs from HC and both patients (P1 and P2) revealed comparable levels of *TREX1* mRNA, suggesting that the decreased TREX1 protein levels in the patients are not due to transcriptional defects but rather a post-transcriptional impairment (**Figure 2C**).

**Figure 3:**
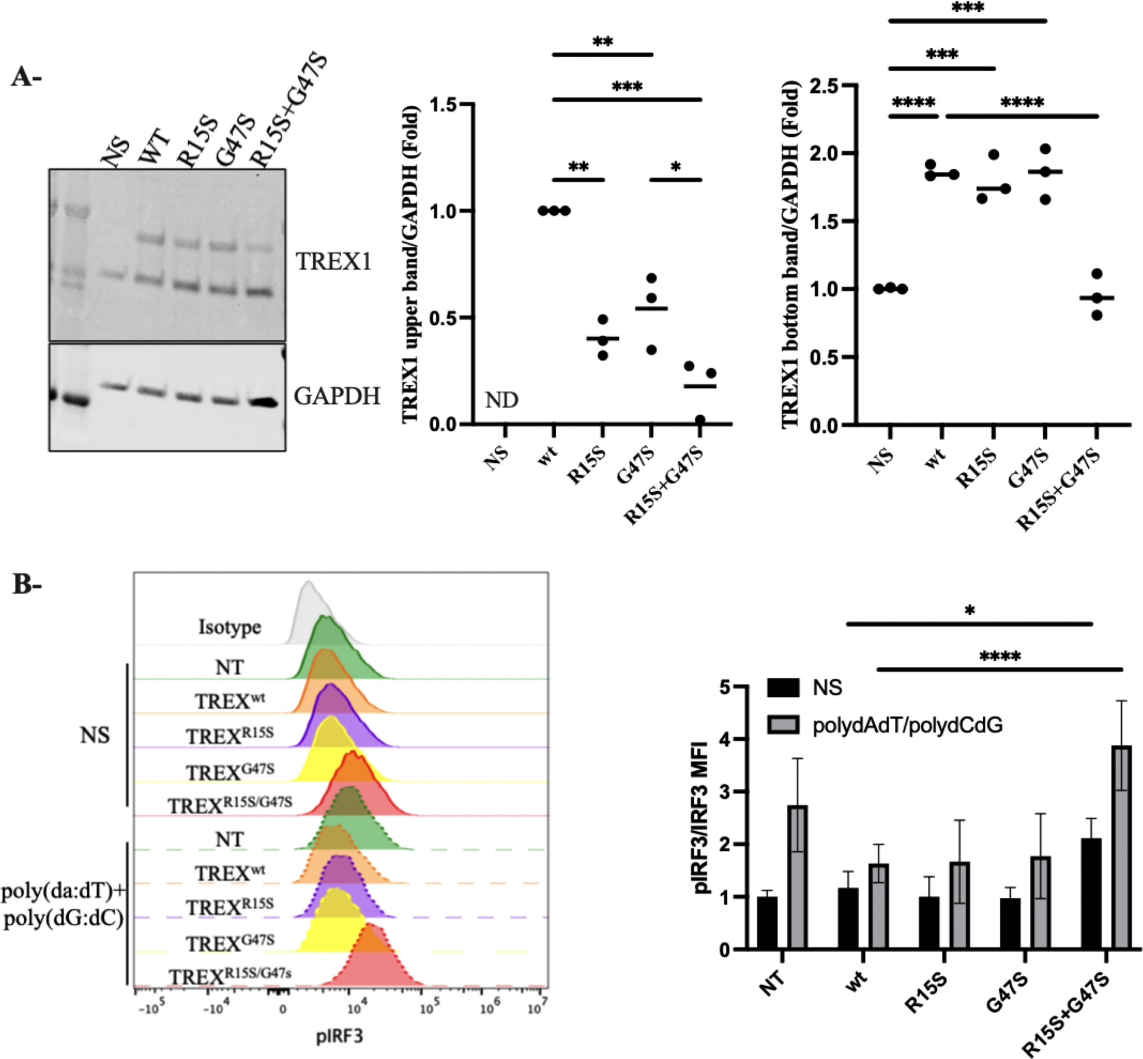
Functional Analysis of TREX1 Variants **(A)** Western Blot determination of TREX1 in fibroblasts transfected with TREX1^wt^, TREX^R15S^, TREX^G47S^ or TREX^R15S/G47S^. GAPDH served as a loading control. The two isoforms of TREX1 were quantified separately. The upper band corresponds to the 369-amino acid isoform, while the lower band represents the 314-amino acid isoform. Densitometry for protein levels was performed and normalized to GAPDH levels. **(B)** Phosphorylation of IRF3 were determined in Flow cytometry in fibroblast transfected with TREX1^wt^, TREX^R15S^, TREX^G47S^ or TREX^R15S/G47S^. Cells were stimulated with poly(dA:dT) (1ug/ml) and poly(dG:dC) (1ug/ml) for 30 min. MFI, mean fluorescence intensity. Data represent the means from triplicates, and results are representative of at least three independent experiments. *p<0.1234, **p<0.0332, ***p<0.0021, ****p<0.0001.

To analyze the impact of the identified *TREX1* variants on protein expression, we constructed a plasmid encoding the longest TREX1 isoform, corresponding to a 369-amino-acid protein (UniProt Q9NSU2-3). This plasmid containing the wild-type TREX1 sequence (NM_016381, Origene RC214869) was used as a template to introduce the two variants, R15S and G47S, for subsequent functional analyses (**Figure 2A**). We transfected fibroblast cells with constructs expressing wild-type TREX1 (TREX1^WT^), each variant individually (TREX1^R15S^ or TREX1^G47S^), or in combination (TREX1^R15S/G47S^). Immunoblot analysis (**Figure 3A**) revealed the presence of two distinct bands, likely corresponding to two isoforms of TREX1. The upper band predominantly represents the longer isoform (369 amino acids), while the lower band corresponds to the shorter isoform (314 amino acids), which is endogenously expressed in fibroblasts. Quantification of the individual bands demonstrated a more pronounced reduction in TREX1 expression when both mutations (R15S and G47S) were present concurrently, compared to single mutations or the wild-type condition. Despite the use of an artificial expression system with a different TREX1 isoform, these findings demonstrate that it is the simultaneous presence of both mutations that result in the near-absence of protein observed in patient-derived cells.

Because loss of TREX1 function augments IRF3-dependent type I IFN production (20), we assessed the functional consequences of these TREX1 mutations singly and in combination. Fibroblasts transfected with the above constructs (TREX1^WT^, TREX1^R15S^, TREX1^G47S^, or TREX1^R15S/G47S^) were stimulated with poly(dA:dT) and poly(dG:dC), synthetic double-stranded DNA (dsDNA) analogs that mimic viral DNA, to activate the cGAS-STING pathway and phospho-IRF3 was measured by flow cytometry (**Figure 3B**). Significantly higher levels of IRF3 phosphorylation were noted in fibroblasts simultaneously bearing both TREX1 variants (TREX1^R15S/G47S^) compared to individual variants (TREX1^R15S^ or TREX1^G47S^) or TREX1^WT^. These data suggest that it is the combination of both TREX1 R15S and G47S mutations that drive hyperactivation of the cGAS-STING pathway, leading to increased production of type I interferons.

### 3.4 Immune evaluation and ISG signature

**Figure 4:**
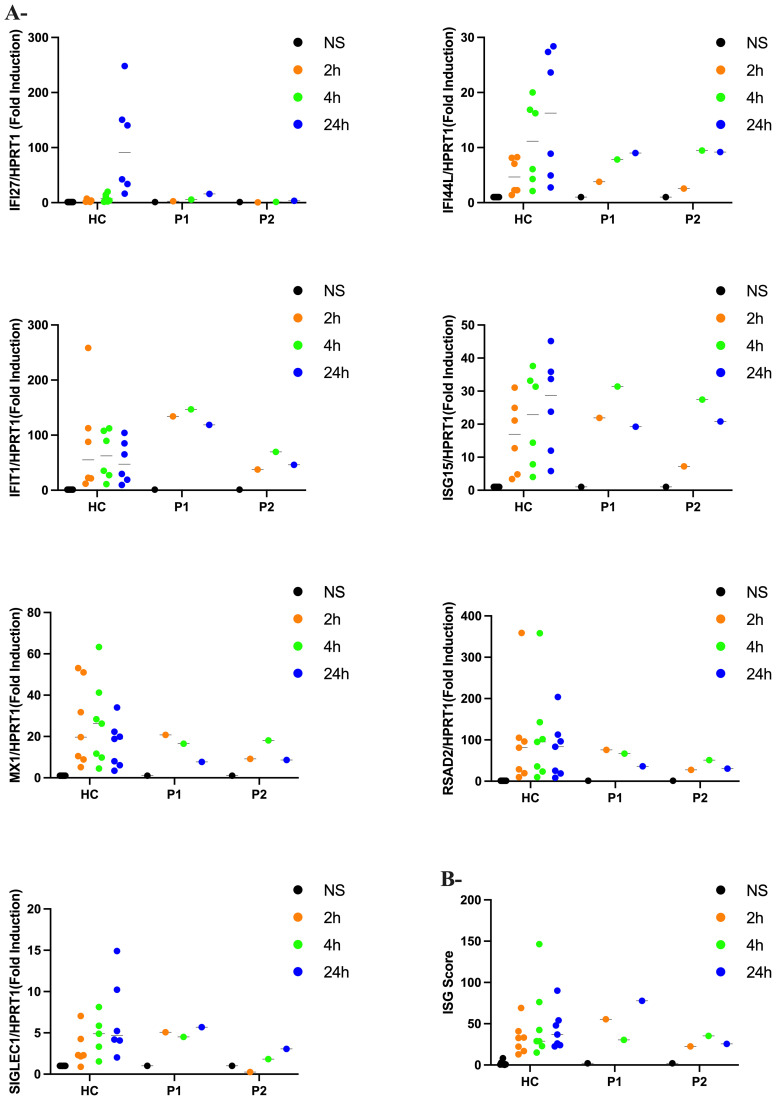
Interferon scores in patients and controls **(A)** qPCR of a panel of seven ISGs in PBMC measured in 2 patients with Aicardi-Goutières syndrome (P1 and P2) and controls (HC) stimulated with IFN-a (100pg/ml) at 2h, 4h and 24h. Horizontal black bars show the median RQ value for each probe in each group RQ is equal to 2^-ΔΔCt^. Data represent the means from triplicates, and results are representative of at least three independent experiments **(B)** Interferon score in all patients and controls calculated from the median fold change in relative quantification value for a panel of six interferon-stimulated genes. Analyzed with one-way ANOVA with Dunnett’s multiple comparison test.

In biomarker analysis of patients’ sera, we observed markedly elevated levels of IFN-α in P1and P2 at ages 4 and 3 years, respectively (**Table 2**). In contrast, IFN-α levels were normal in patient 3 (P3) at the age of 15 years. Additionally, cerebrospinal fluid (CSF) analysis revealed elevated levels of neopterin and tetrahydrobiopterin (pteridine-derivative immune activation biomarkers that can be induced by IFN-α) in P2 and P3 at ages 3 and 15 years, respectively. These findings prompted us to evaluate the ISG signature from IFN-α – stimulated PBMC. Quantitative PCR analysis (**Figure 4A**) revealed no statistically significant differences between HC and patients (P1 and P2) in the expression levels of each ISG and of the calculated ISG score (**Figure 4B**).

**Table 2:** Immune laboratory findings for Patients 1, 2 and 3.

Lab Marker	Patient 1	Patient 2	Patient 3	Reference range
Interferon alpha (serum)	**18**	**13**	<2	<=2 pg/mL
Plasma IL-1 beta	<4	–	–	<=4 pg/mL
Plasma IL-2	<6	-	-	<=8 pgm/mL
Plasma IL-4	<16	–	–	<=19 pg/mL
Plasma IL-5	<5	-	-	<=5 pg/mL
Plasma IL-6	**9**	–	–	<=8 pg/mL
Plasma IL-8	**42**	-	-	<=9 pg/mL
Plasma IL-10	<6	–	–	<=7 pg/mL
Plasma IFN gamma	1	-	-	<= 1 pg/mL
Plasma TNF alpha	<2	–	–	<=3 pg/mL
Plasma GM-CSF	<3	-	-	<=4 pg/mL
Plasma CXCL9	437	–	–	<=647 pg/mL
CSF Neopterin	-	**254**	**68** (8-28)	7-65 nmol/L
CSF Tetrahydrobiopterin	–	**114**	**61** (10-30)	18-50 nmol/L
C3	**2.014**	1.298	1.062	0.773 – 1.765
C4	0.21	0.167	0.157	0.11- 0.419
CH50	92.7 U/mL	50.09 U/mL	90.71	No pediatric reference
IgG	**22.34**	**11.71**	8.93	4.85 – 11.6 g/L
IgA	1.35	0.94	1.94	0.14 – 2.12
IgM	1.3	1.47	0.61	0.26 – 1.55
IgE	11	9	4	0-60 IU/mL
CD3+	1702	1628	988	782-2585 cells/mm3
CD4+	965	1133	585	408-1538 cells/mm3
CD8+	635	425	351	244-986 cells/mm3
%TCRab DNTC	**3.70%**	1.8	1	0-2%
CD19+	660	590	195	110-1400 cells/mm3
CD3-CD16+CD56+	178	118	117	70-720 cells/mm3
%CD4+CD45RA+	-	76	59	39-83% of CD4+ T cells
%CD8+CD45RA+	–	78	71	65- 97% of CD8+ T cells
%CD4+RTE	-	64.5	44.9	19.4-68% of CD4+ Tcells
%CD19+CD27+ memory B cells	–	**2%**	21	19-36% of B cells
%CD19+CD27+IgM+IgD+ marginal zone B cells	-	**1%**	**7**	8-22% of B cells
%CD19+CD27+IgM-IgD- switched memory B cells	–	**0.8**	11.2	3.5 – 14.4% of B cells

Bold values are outside the normal range.

## 4 Discussion

Aicardi-Goutières syndrome (AGS) is a monogenic type I interferonopathy characterized by dysregulated immune responses to endogenous nucleic acids. In this study, we identified two homozygous mutations in the *TREX1* gene (c.-26-1G>A and c.-26-95G>T, based on NM_033629.6) in two Inuit siblings from the Sanikiluaq region of North of Quebec, presenting with severe developmental delay, neurodegeneration, and systemic inflammation. These patients’ geographical and cultural background is noteworthy, as AGS is allelic with “Cree encephalitis,” which was first reported among the Cree people of the James Bay region of northwestern Quebec. The Sanikiluaq archipelago, located at an intermediate latitude between the Arctic (Inuit) and James Bay (Cree) regions, might have a founder effect due to geographical isolation, admixture, and endogamy. This could potentially explain the morbidity observed in other family members who were not evaluated in this study. Further collaboration with local officials may help assess the prevalence of this allele in the region, with important implications for personalized health strategies.

Initial genetic studies were only partially revealing. However, immunoblot analysis of LCLs from P1 revealed a profound reduction in TREX1 protein levels; this prompted re-evaluation of the genetic data, which identified the presence of two homozygous variants. Overexpression experiments in fibroblast cells demonstrate that the simultaneous presence of both variants is required to mirror the markedly reduced TREX1 protein level observed in patient-derived cells, emphasizing the importance of molecular validation of identified genetic variants. Further support for this synergistic effect is that the aberrant increase in phosphorylated (activated) IRF3 is observed with both variants, accounting for the dysregulated type I interferon signaling, and hence the clinical features of this interferonpathy. Our findings expand the genetic spectrum underlying AGS and highlight, for the first time to our knowledge, the compound effect of variants on TREX1 expression and regulation of the cGAS-STING pathway.

Interestingly, despite elevated plasma IFN-α levels, PBMCs from P1 and P2 did not exhibit an elevated ISG signature that has been characteristically seen in other patients with AGS. This phenomenon of a negative ISG score in patients with AGS has been reported before, although in a minority of patients and particularly in those bearing *RNASEH2B* mutations (3). As well, there is heterogeneity in individual ISG genes to measure and integrate into a score, although we have used a panel that has been validated by others (3, 27). Circulating autoantibodies to type I IFN may downregulate ISG expression (3), although neither P1 or P2 had such demonstrable autoantibodies. In the Inuit, a deleterious p.S53P mutation in IFNAR2, a subunit of the type I IFN receptor, has been reported with high frequency (28) (1.25%), although these children did not genetically have autosomal recessive IFNAR2 deficiency. Persistent type I IFN activity (indicated by elevated plasma IFN-α levels and CSF pteridines), despite a normal ISG profile, suggests that the brain damage in these two children with AGS may not be directly caused by the specific ISGs measured. and that alternate pathophysiological processes are at play. Indeed, chronic inflammation (marked by dysregulated neopterin) may contribute to brain calcification via oxidative stress and microvascular damage, while abnormal tetrahydrobiopterin (BH4) can lead to deranged neurotransmitter levels and altered neurodevelopment, Interestingly, atypical phenylketonuria caused by BH4 deficiency can also have abnormal neurotransmitter levels, neurodevelopmental delays, and brain calcification (29).

Altogether, by identifying compound homozygous mutations in TREX1. Our work expands the genetic basis for AGS. In this population, these findings have potential public health implications. More broadly, a similar genetic phenomenon may underlie other inborn errors of immunity.

## Data Availability

The datasets presented in this study can be found in online repositories. The names of the repository/repositories and accession number(s) can be found in the article/**Supplementary Material**.
