# Supplementary material for: Two rare mutations in homozygosity synergize to silence TREX1 in Aicardi-Goutières syndrome
Source: Front Immunol. 2025 Feb 21;16:1557632. doi: 10.3389/fimmu.2025.1557632 (PMC11885508; doi:10.3389/fimmu.2025.1557632)
Supplement: Supplementary file 1 [file DataSheet1.docx]

**Supplemental Information: Clinical Details**

**Patient 1 Immunization History and Immune Evaluation**

Patient 1 (P1) received 4 doses of the 13-valent pneumococcal conjugate vaccine (PCV13) at 2, 6, 8, and 16 months old. She then received a dose of pneumococcal polysaccharide vaccine (Pneumovax) at 26 months old, in accordance with the territorial immunization schedule.

At 3 years and 7 months old, baseline semi-quantitative pneumococcal titers were assessed as part of her initial immune evaluation. Despite full and recent immunization and recurrent pneumonia episodes, her pneumococcal capsular polysaccharide IgG was 10.273 mg/L, and pneumococcal capsular polysaccharide IgG2 was 1.782 mg/L. Other vaccine titers, including tetanus and diphtheria, were low, while measles and rubella titers were undetectable, despite prior vaccinations. She received a second diagnostic booster dose of Pneumovax at 3 years and 7 months old. Post-booster semi-quantitative pneumococcal capsular polysaccharide IgG rose to 262.462 mg/L, pneumococcal capsular polysaccharide IgG2 22.349 mg/L. Post-booster serotype specific testing was concurrently sent, demonstrating a protective response to 18/22 serotypes tested, and 6/10 exclusively found in the polysaccharide vaccine. This was considered a normal/reassuring response. Pre-booster serotype-specific data was unavailable for comparison.

At 5 years and 2 months old, semi-quantitative pneumococcal titers were rechecked without an intervening booster. Results indicated a decline in pneumococcal capsular polysaccharide IgG to 5.833 mg/L, while pneumococcal capsular polysaccharide IgG2 was detected but not quantifiable (<0.61 mg/L). Measles IgG was also undetectable at this time, despite three doses of the MMR vaccine.

Table S1: Patient 1 Immunization Results

|  | Baseline* | 6 weeks after boosters (MMR, TdaP, Pneumovax) | Age 5** |
| --- | --- | --- | --- |
| Measles IgG | **Not detected** | Detected | **Not detected** |
| Mumps IgG | Detected | Detected | Detected |
| Rubella IgG | **Not detected** | Detected | Detected |
| Tetanus toxin IgG | **0.111 IU/mL** | >5 IU/mL | 2.260 IU/mL |
| Diphtheria toxin IgG | **0.05 IU/mL** | >2 IU/mL | 1.373 IU/mL |
| Streptococcus pneumoniae capsular polysaccharide IgG | **10.273 mg/L** | 262.462 mg/L | **5.833 mg/L** |
| Streptococcus pneumoniae capsular polysaccharide IgG2 | **1.782 mg/L** | 22.349 mg/L | **Detected, not quantifiable** |

*Baseline: At 3 years 7 months old - After 4 doses of PCV13, 4 doses of DtaP-IPV containing vaccine, 1 dose of Pneumovax, 2 doses of MMRV

**Age 5, following a total of 4 doses of PCV13, 2 doses of Pneumovax, 5 doses of TdaP-IPV vaccine, 3 doses of MMRV. Bold values are outside the normal range.

**Patient 2 Immunization and Immune Response**

Patient 2 (P2) had low baseline vaccine titers when tested at 2 years and 11 months old. Measles, mumps, and rubella IgG were undetectable. Tetanus toxin IgG was 0.111 IU/mL, and pneumococcal capsular polysaccharide IgG was 40.677 mg/L, while pneumococcal capsular polysaccharide IgG2 was 10.181 mg/L.

By age 4, following a third MMR and fifth TdaP booster, measles, mumps, and rubella IgG remained undetectable. Tetanus and diphtheria toxin IgG were slightly improved but remained suboptimal. Pneumococcal capsular polysaccharide IgG had declined to 4.193 mg/L, and pneumococcal capsular polysaccharide IgG2 was detected but not quantifiable.

Table S2: Patient 2 Immunization Results

|  | Baseline* | Age 4** |
| --- | --- | --- |
| Measles IgG | **Not detected** | **Not detected** |
| Mumps IgG | **Not detected** | **Not detected** |
| Rubella IgG | **Not detected** | **Not detected** |
| Tetanus toxin IgG | **0.111 IU/mL** | 0.244 IU/mL |
| Diphtheria toxin IgG | **Not done** | 0.266 IU/mL |
| Streptococcus pneumoniae capsular polysaccharide IgG | 40.677 mg/L | 4.193 mg/L |
| Streptococcus pneumoniae capsular polysaccharide IgG2 | 10.181 mg/L | **Detected, not quantifiable** |

*Baseline: Age 2 years 11 months, after having received 4 doses of DtaP-IPV containing vaccine, 4 doses of PCV13, 1 dose of Pneumovax, 2 doses of MMR. **Age 4: <1 year after having received 3^rd^ MMR and 5^th^ TdaP boosters. Bold values are outside the normal range.

**Patient 3 Clinical History and Immunization**

Patient 3 (P3) is a female born at term after an uncomplicated pregnancy and delivery who was noted to have gross motor developmental delay and central hypotonia at four months old. By age 2, she was diagnosed with global developmental delay (GDD) and spastic diplegic cerebral palsy (CP). She did not ambulate independently until age 4. Brain MRI showed T2-weighted hyperintensities in the periventricular white matter. Progressive dystonia began at age 15, prompting further investigations, but a repeat MRI showed no new findings. To date, she has not had severe or recurrent respiratory tract infections, respiratory failure, or lupus-like symptoms.

P3’s baseline immunization serology showed detectable measles IgG, but undetectable mumps and rubella IgG. Tetanus toxin IgG was 0.127 IU/mL, and pneumococcal capsular polysaccharide IgG was 29.888 mg/L, with pneumococcal capsular polysaccharide IgG2 at 10.634 mg/L. Her full vaccine schedule was not available for review.

Table S3: Patient 3 Immunization Results

|  | Baseline* |
| --- | --- |
| Measles IgG | Detected |
| Mumps IgG | **Not detected** |
| Rubella IgG | **Not detected** |
| Tetanus toxin IgG | **0.127 IU/mL** |
| Diphtheria toxin IgG | 1.476 IU/mL |
| Streptococcus pneumoniae capsular polysaccharide IgG | 29.888 mg/L |
| Streptococcus pneumoniae capsular polysaccharide IgG2 | 10.634 mg/L |

*Vaccine schedule not available for review. Bold values are outside the normal range

**Patient 4 Clinical History and Immunization**

Patient 4 (P4), a sister to P3, presented at four months old with hypotonia, developmental delay, seizures, and severe laryngomalacia. Her brain MRI revealed increased T2 and decreased T1 signal intensity in the deep and periventricular white matter, with abnormalities extending into the subcortical white matter in the parasagittal regions with associated gyral swelling. At 20 months, she presented with respiratory failure and died after admission to the Pediatric Intensive Care Unit.

Interferon score was not obtained for P3, and P4 died before immunology or autoimmune evaluation could be assessed.

**Baricitinib Treatment Consideration**

Parents were reluctant to begin JAK inhibitor therapy for a variety of reasons, probably including a degree of mistrust of the medical system stemming from longstanding and historical abuse of First Nations, Inuit and Metis patients from the medical system. P1’s mother expressed hesitation about offering an “experimental” therapy for her daughter’s condition, especially knowing that the clinical benefit was uncertain given limited published studies. Further, she expressed concerns about risk of severe infection that was disclosed by the medical team, including fatal infection, particularly since her daughter was already repeatedly and frequently admitted with (presumed) respiratory tract infection. When the family initially agreed to start Baricitinib as a trial, the patient was admitted for respiratory failure after having only used a few days of the medication. Attributing the admission to a possible effect of the Baricitinib, the family was then reluctant to resume the medication upon discharge.

Following subsequent radiographic and clinical progression of P1s respiratory condition, and ongoing demonstration of impaired humoral immunity, the family decided to trial Baricitinib again, along with simultaneous immune globulin replacement. The family is still considering Baricitinib trial for P2, who appears to be less severely affected compared to her sibling at this time.

Table S4: ANA, ANA patterns, anti-dsDNA results in P1-4

|  | Patient 1 | Patient 2 | Patient 3 | Patient 4 |
| --- | --- | --- | --- | --- |
| ANA Ab Hep20/10 | 1:160 nuclear Ab speckled  1:80 cytoplasmic antibody speckled | 1:80 nuclear Ab speckled  Negative mitotic pattern and cytoplasmic pattern | Negative nuclear, cytoplasmic, mitotic pattern | Not done |
| Anti-dsDNA | <10 IU/mL | <10 Iu/mL | <10 IU/mL | Not done |
| C-ANCA | Indeterminate (<1:10 titer) | negative | negative | Not done |
| P-ANCA | Indeterminate (<1:10 titer) | Negative | Negative | Not done |
